# Genetic variability and response to sertraline in pediatric populations: a review on pharmacogenetics, pharmacokinetics, and the risk of adverse events

**DOI:** 10.3389/frcha.2026.1731268

**Published:** 2026-02-17

**Authors:** Jesús Alonso Gándara-Mireles, Verónica Loera Castañeda, Ismael Lares-Asseff, Julio Cesar Grijalva Ávila, Maria Magdalena Rosales Ramos, Ignacio Villanueva Fierro, Leslie Patrón Romero, Horacio Almanza Reyes

**Affiliations:** 1 CIIDIR-Durango Unit, National Polytechnic Institute, Genomics Academy, Durango, Mexico; 2 Latin American Network for the Implementation and Validation of Pharmacogenomic Clinical Guidelines (RELIVAF-CYTED), Santiago, Chile; 3 Faculty of Medicine and Nutrition, Juárez University of the State of Durango, Durango, Mexico; 4 School of Medicine and Psychology, Autonomous University of Baja California, Tijuana, Mexico

**Keywords:** anxiety disorders, pediatric population, pharmacogenetics, pharmacokinetics, sertraline

## Abstract

Anxiety disorders in the pediatric population represent a highly prevalent mental health concern whose pharmacological management has been consolidated through the use of selective serotonin reuptake inhibitors (SSRIs). Among these agents, sertraline is one of the most frequently prescribed; however, its efficacy and safety in children and adolescents exhibit substantial interindividual variability, largely attributed to clinical, physiological, and genetic factors. This review aimed to analyze the current evidence on the efficacy, safety, and optimization strategies for sertraline therapy in pediatric patients, with a particular focus on pharmacokinetic and pharmacogenetic determinants that modulate therapeutic response. Available evidence indicates that genetic variants in CYP2C19, CYP2D6, and ABCB1 significantly influence hepatic metabolism, plasma exposure, and drug tolerability. These differences support the integration of pharmacogenetic testing as a clinical tool to individualize dosing and prevent adverse effects. In addition, population pharmacokinetic modeling has emerged as a valuable approach to design personalized therapeutic regimens, especially for patients with medical comorbidities or atypical metabolic profiles. In conclusion, the integration of clinical, genetic, and pharmacokinetic information into pediatric psychiatric practice may facilitate the advancement of precision medicine, promoting safer, more effective, and individualized sertraline-based treatments for anxiety disorders in children and adolescents.

## Background

Anxiety disorders constitute one of the most frequent psychiatric conditions during childhood and adolescence, exerting a substantial impact on patients' emotional, academic, and social development (1–3). Globally, it is estimated that approximately 4% of children experience some form of anxiety disorder, although prevalence varies according to sociodemographic factors and regional health system differences (4). In Mexico, the *National Psychiatric Epidemiology Survey* reported that about 8.8% of the population has experienced an episode of anxiety during their lifetime, ranking anxiety disorders among the leading causes of psychiatric morbidity particularly in women and pediatric populations (5). This high prevalence has driven the search for effective and safe pharmacological treatments tailored to the individual needs of each patient. Among the available therapeutic options, sertraline, a SSRI, has been consolidated as a first-line treatment for anxiety disorders in children and adolescents (6, 7). Its mechanism of action involves inhibition of presynaptic serotonin reuptake, thereby increasing synaptic serotonin availability and enhancing serotonergic neurotransmission a key process in the regulation of mood and anxiety (8). Despite its widespread use, clinical response to sertraline exhibits substantial interindividual variability in both efficacy and tolerability (9). While some patients achieve an optimal therapeutic response with standard doses, others show minimal clinical improvement or develop adverse effects (AEs) that compromise treatment adherence.

This therapeutic heterogeneity underscores the importance of understanding the biological factors that modulate pharmacological response, among which pharmacogenetics plays a central role (10, 11). This discipline examines how genetic variations influence drug response. In the case of sertraline, multiple cytochrome P450 (CYP) enzymes are involved in hepatic metabolism, primarily CYP2C19, CYP2D6, and CYP2C9 (12–14). These enzymes harbor single nucleotide variants (SNVs) that alter catalytic activity, classifying individuals as ultrarapid, extensive, intermediate, or poor metabolizers. Variants that reduce enzymatic activity can lead to elevated plasma concentrations, increasing the risk of toxicity and AEs, whereas those associated with enhanced activity accelerate drug clearance, potentially reducing clinical efficacy (12, 14). Recent studies have demonstrated that patients carrying specific genotype combinations require individualized dose adjustments to achieve optimal therapeutic concentrations while minimizing AEs. Kee et al. (2023) (12) reported that carriers of the *CYP2D6*4* and *CYP2C19*2* alleles exhibited greater plasma exposure to antidepressants and an increased likelihood of AEs, suggesting the need for dose reduction in these individuals. Similarly, Huddart et al. (2020) (15) conducted a detailed analysis of the pharmacokinetic pathway of sertraline using the *PharmGKB* database, identifying that interindividual variability in plasma levels is strongly determined by differential expression of CYP2C19 and CYP2D6 enzymes. Their findings emphasize that such genetic variation can significantly influence antidepressant efficacy and safety, particularly in younger populations. In the pediatric context, this information acquires special clinical relevance since children exhibit physiological differences compared to adults, including developing hepatic metabolism, variable drug bioavailability, and differences in volume of distribution. These factors, combined with genetic variability, render the pharmacokinetics of sertraline particularly complex and difficult to predict with precision (16, 17).

Although this review places particular emphasis on Latin American and Mexican pediatric populations due to the limited availability of local pharmacogenetic data, it is important to acknowledge that similar evidence gaps exist in other regions worldwide. Pediatric populations from Asia, Africa, the Middle East, and other low- and middle-income settings remain markedly underrepresented in pharmacogenetic and population pharmacokinetic studies of antidepressants. These populations face comparable challenges, including limited access to genetic testing, reduced participation in clinical trials, and interethnic variability in allele frequencies that may influence sertraline exposure, efficacy, and safety (18–20).

Within this framework, population pharmacokinetic (PopPK) modeling has emerged as an essential tool for integrating clinical and pharmacogenetic data. This approach quantifies interindividual variability and identifies covariates that influence drug absorption, distribution, metabolism, and elimination (9–22). PopPK modeling enables estimation of individual pharmacokinetic parameters from limited clinical data, simulation of diverse dosing scenarios, and the design of personalized therapeutic regimens based on genetic and physiological profiles. Several international studies have applied PopPK models to describe sertraline pharmacokinetics in pediatric patients. Stoiljkovic et al. (2023) (23) developed a pharmacokinetic model in patients with depression and demonstrated that sertraline clearance is significantly influenced by serum concentrations of its metabolite *N-desmethylsertraline*, creatinine clearance, and daily dose. These findings highlight the utility of population modeling in adjusting doses according to renal function and other individual parameters. Likewise, Poweleit et al. (2023) (17) analyzed the pharmacokinetics of sertraline and escitalopram in a pediatric cohort from the northern United States, reporting that carriers of the *CYP2C19*2* allele had significantly higher plasma exposure compared to normal metabolizers. The study concluded that these patients could benefit from dose reductions to avoid toxicity and maintain therapeutic levels. More recently, Zhang et al. (2024) (21) applied a similar PopPK approach in Chinese pediatric patients, demonstrating that the combined use of clinical and genetic factors including *CYP2C19* metabolizer status allowed accurate prediction of sertraline plasma concentrations. Their model enabled effective dose individualization, optimizing therapeutic response and reducing the risk of adverse events.

### Background and rationale

In Mexico, the increasing incidence of anxiety disorders among school-aged children has become a growing concern for mental health professionals. Despite the widespread use of sertraline in this population, few studies have specifically examined its efficacy, pharmacokinetics, and safety in Mexican pediatric patients. This lack of local evidence limits the extrapolation of international findings to the national context, where significant clinical challenges persist regarding variability in therapeutic response, the occurrence of adverse events, and treatment adherence (22). Therefore, it is imperative to generate context-specific evidence that characterizes the pharmacokinetics of sertraline in Mexican pediatric patients, identifies the most prevalent genetic variants, and explores their associations with relevant pharmacokinetic and clinical parameters. In parallel, similar limitations have been reported in pediatric populations from other regions, including Asia, Africa, and the Middle East, where pharmacogenetic data remain scarce or are derived from small, heterogeneous cohorts (18–20). These shared evidence gaps highlight that the challenge of extrapolating pharmacogenetic findings from predominantly adult or European populations is not unique to Mexico, but rather reflects a broader global issue in pediatric psychopharmacology.

**Figure 1:**
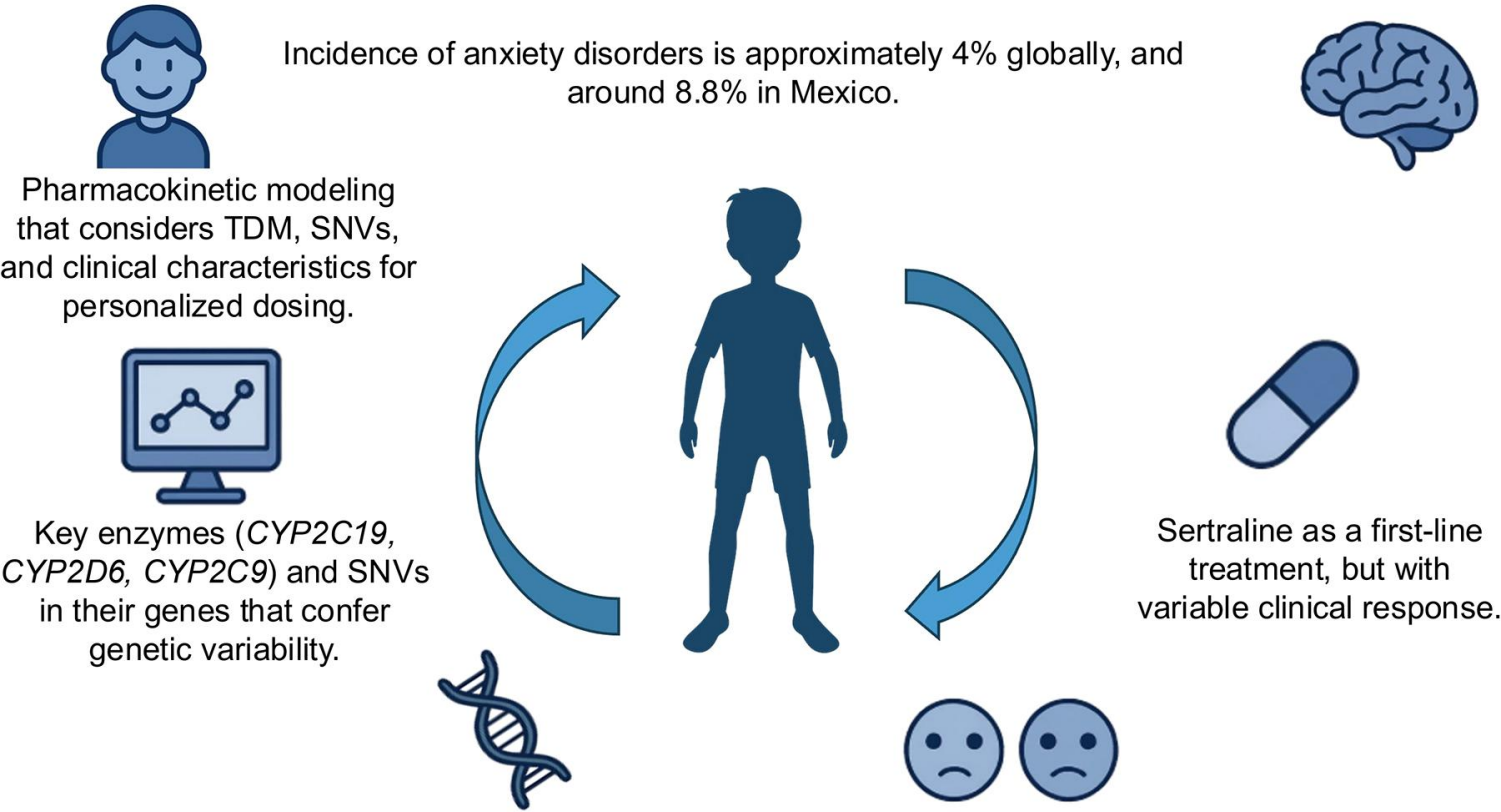
Clinical, genetic, and pharmacokinetic determinants of response to sertraline in pediatric patients with anxiety disorders. Schematic representation of the main clinical, genetic, and pharmacokinetic determinants that influence therapeutic response to sertraline in the pediatric population.

The present review aims to synthesize and analyze the available evidence on the impact of pharmacogenetics on the pharmacokinetics and safety of sertraline in children and adolescents with anxiety disorders. It examines the main genes involved in sertraline metabolism, the frequency and functional relevance of their variants, PopPK models applicable to pediatric cohorts, and the clinical implications of these findings for treatment optimization. This analysis seeks to provide a scientific and conceptual framework supporting the incorporation of pharmacogenomic tools into pediatric psychiatry and to promote the development of personalized, safe, and effective pharmacological strategies for managing anxiety disorders in this age group (Figure 1).

### Mechanism of action and therapeutic target of sertraline

**Figure 2:**
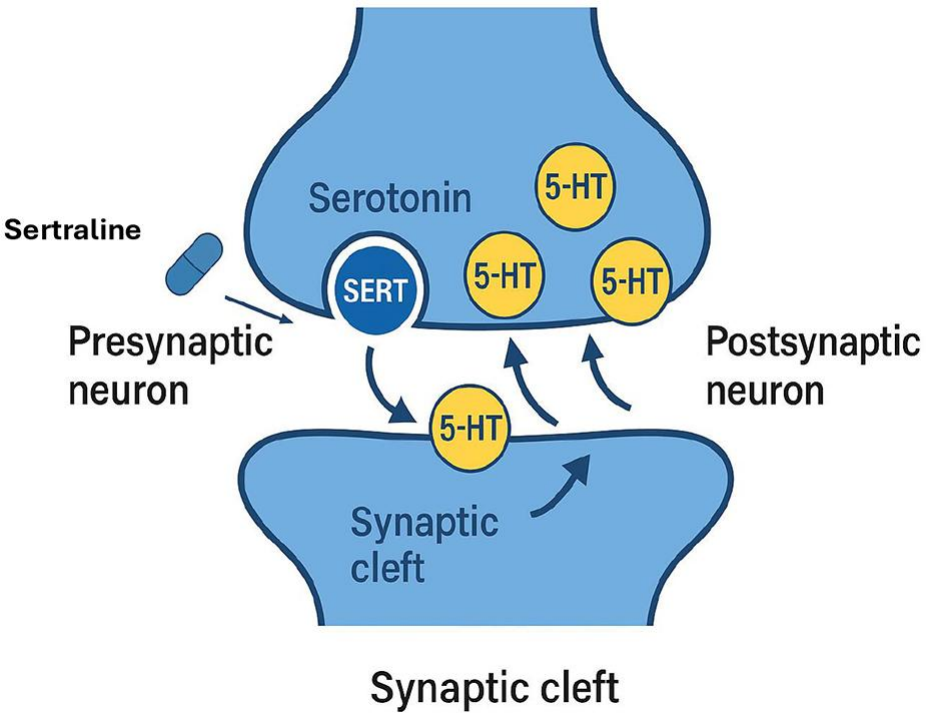
Synaptic mechanism of action of sertraline. Sertraline acts by selectively inhibiting the serotonin transporter (SERT) located in the presynaptic membrane, thereby preventing the reuptake of serotonin (5-HT) from the synaptic cleft into the presynaptic neuron. This action increases the availability of serotonin in the synaptic cleft, promoting serotonergic neurotransmission to the postsynaptic neuron, which contributes to the improvement of anxiety and depressive symptoms.

Sertraline is a SSRI widely employed in the treatment of various mood and anxiety disorders in both adults and children (24). Its use in pediatric and adolescent populations has increased steadily due to its favorable safety profile and demonstrated efficacy in reducing anxious and depressive symptoms (25). The mechanism of action of sertraline involves selective inhibition of the serotonin transporter (SERT), a membrane protein located on presynaptic serotonergic neurons. This transporter is responsible for the reuptake of serotonin (5-hydroxytryptamine, 5-HT) released into the synaptic cleft back into the presynaptic neuron for recycling or degradation (26). By blocking this process, sertraline increases serotonin availability in the synaptic space, enhancing serotonergic neurotransmission and thereby contributing to the regulation of mood, anxiety, and affective processing. Sustained elevation of synaptic serotonin levels has been correlated with gradual clinical improvement in various psychiatric conditions (27, 28) (Figure 2). From a pharmacodynamic perspective, sertraline exhibits high affinity for the SERT and low affinity for other receptor systems, including adrenergic, cholinergic, and dopaminergic receptors (29–31). This selectivity accounts for its lower incidence of collateral adverse effects compared with tricyclic antidepressants and earlier-generation agents.

Preclinical studies in animal models have shown that sertraline not only increases extracellular serotonin but also dopamine levels in specific brain regions, such as the nucleus accumbens and striatum, which may contribute to improvements in symptoms like anhedonia and loss of motivation, particularly in depressive disorders (32). Although these dopaminergic effects have not yet been conclusively demonstrated in humans through direct methods such as microdialysis or functional neuroimaging, experimental evidence suggests a complementary dopaminergic role in the therapeutic response to sertraline, highlighting opportunities for future clinical research. In pediatric patients, the therapeutic goal of sertraline is to reduce anxiety-related symptoms, including excessive worry, restlessness, difficulty sleeping, irritability, and associated somatic manifestations (33). In disorders such as generalized anxiety disorder (GAD), obsessive-compulsive disorder (OCD), social anxiety disorder, and separation anxiety disorder, sertraline acts by restoring neurochemical balance within emotion-regulation circuits, thereby facilitating better school, social, and family adaptation (34).

Furthermore, sertraline has demonstrated efficacy in treating common comorbidities of childhood, such as major depressive disorder (MDD) and OCD (35). Systematic reviews and meta-analyses have identified sertraline as one of the SSRIs with the strongest evidence supporting both efficacy and safety in pediatric populations, especially when combined with psychotherapeutic interventions such as cognitive-behavioral therapy (CBT). In this context, treatment optimization with sertraline regarding dose titration, onset of action, tolerability, and risk of adverse events depends not only on general clinical factors but also on individual characteristics such as age, body weight, degree of hepatic maturation, and, increasingly recognized, the pharmacogenetic profile of the patient (36, 37).

### Therapeutic applications of sertraline in the pediatric population

Since its initial approval by the U.S. Food and Drug Administration (FDA) in 1991 for the treatment of major depressive disorder (MDD) in adults, sertraline has progressively expanded its therapeutic spectrum to encompass a variety of psychiatric conditions in both adult and pediatric populations. It is currently approved for the management of anxiety disorders, obsessive-compulsive disorder (OCD), post-traumatic stress disorder (PTSD), premenstrual dysphoric disorder (PMDD), and MDD (38).

Anxiety disorders in the pediatric population

The primary indication for sertraline in children and adolescents is the treatment of anxiety disorders, where it has demonstrated efficacy in reducing clinical symptoms and improving psychosocial functioning. The most prevalent conditions include generalized anxiety disorder (GAD), social anxiety disorder, and separation anxiety disorder, all of which are common during childhood and can substantially interfere with emotional, academic, and social development (8). Evidence from multicenter clinical trials supports the efficacy of sertraline, either as monotherapy or in combination with psychotherapy. The Child/Adolescent Anxiety Multimodal Study (CAMS), published in *The New England Journal of Medicine*, showed that the combination of sertraline plus cognitive-behavioral therapy (CBT) was significantly more effective than either intervention alone for treating GAD, social anxiety disorder, and separation anxiety disorder in patients aged 7–17 years (8). These findings reinforce the role of sertraline as a first-line pharmacological option within a multimodal therapeutic framework.

2. Obsessive-Compulsive Disorder (OCD)

Sertraline is one of the few SSRIs formally approved for the treatment of pediatric OCD. Clinical evidence indicates that it effectively reduces both intrusive thoughts and compulsive behaviors characteristic of the disorder. Double-blind, placebo-controlled trials have demonstrated significant improvement in scores on the Children's YaleBrown Obsessive-Compulsive Scale (CY-BOCS) following sertraline treatment, with a generally mild and manageable adverse event profile (39, 40).

3. Major Depressive Disorder (MDD)

The use of antidepressants in children and adolescents has raised ongoing debate due to the potential risk of suicidal ideation. Nevertheless, sertraline has shown acceptable efficacy and tolerability in moderate-to-severe MDD, particularly in cases where psychotherapy alone has been insufficient. The meta-analysis by Cipriani et al. (2016) (41) identified sertraline as one of the SSRIs with the most favorable balance between efficacy and safety in adolescents with MDD. However, close clinical monitoring during the initial weeks of therapy remains essential, with vigilant observation for affective and behavioral changes.

4. Post-Traumatic Stress Disorder (PTSD)

In children and adolescents exposed to traumatic events such as abuse, natural disasters, or violence, sertraline has demonstrated efficacy in reducing core PTSD symptoms, including hypervigilance, avoidance, irritability, and intrusive recollections. While the strongest evidence originates from adult studies, pilot and open-label pediatric trials support its use in this population, particularly as part of a combined pharmacological psychotherapeutic approach (42, 43).

5. Comorbid and behavioral disorders

Emerging evidence suggests potential benefits of sertraline as a therapeutic alternative for comorbid conditions characterized by anxious or affective symptoms, such as attention-deficit/hyperactivity disorder (ADHD) (44), oppositional defiant disorder (ODD), and autism spectrum disorder (ASD) (45). In these cases, sertraline aims to reduce anticipatory anxiety, cognitive rigidity, and irritability. Although preliminary findings are promising, the current evidence remains heterogeneous, and further controlled studies are required before firm clinical recommendations can be established (46).

6. Use in complex medical and psychiatric contexts

Sertraline has also been successfully used in pediatric patients with chronic medical conditions accompanied by secondary anxious or depressive symptoms, such as type 1 diabetes mellitus, epilepsy, autoimmune diseases, and oncologic conditions (47). Its low potential for pharmacological interactions, owing to moderate inhibition of cytochrome P450 isoenzymes, together with its favorable safety profile during long-term therapy, makes it a reasonable option in these complex clinical contexts, provided that an individualized risk benefit assessment is conducted for each patient.

7. Clinical perspectives according to the pharmacogenetic profile

One of the main challenges in the clinical use of sertraline lies in the marked interindividual variability in treatment response. As discussed in previous sections, genetic variants in key genes such as *CYP2C19*, *CYP2D6*, *CYP2C9*, and *ABCB1* can significantly influence the metabolism, distribution, and elimination of the drug, ultimately modifying its efficacy and the likelihood of adverse reactions (48, 49). According to the Clinical Pharmacogenetics Implementation Consortium (CPIC) guidelines, pediatric patients identified as poor metabolizers of *CYP2C19* may require a 50% reduction in the initial sertraline dose to prevent elevated plasma concentrations and associated toxicity. Conversely, ultrarapid metabolizers might need higher doses or an alternative SSRI if the therapeutic range is not achieved under standard dosing conditions (14, 50). These recommendations highlight the potential of integrating pharmacogenetic profiling into clinical decision-making for sertraline therapy, particularly in cases of therapeutic failure or severe adverse events. Furthermore, the development of population pharmacokinetic models that incorporate genetic, clinical, and demographic data represents a major step toward precision medicine in pediatric psychiatry, enabling more accurate and individualized dosing of selective serotonin reuptake inhibitors (SSRIs) (51, 52).

8. Clinical limitations and considerations

Despite its broad therapeutic applications in pediatric populations, the use of sertraline requires careful clinical consideration due to several limitations. First, many clinical trials lack representative samples of children under 12 years of age, which restricts the generalizability of efficacy and safety outcomes (33, 39). Additionally, adverse effects such as nausea, insomnia, agitation, or suicidal ideation although uncommon necessitate close clinical monitoring, particularly during the first weeks of therapy or following dose adjustments (39). The therapeutic response may take between two and six weeks to become clinically evident (53), emphasizing the need for structured psychoeducation of parents and caregivers to improve adherence and manage expectations. Finally, potential pharmacokinetic and pharmacodynamic interactions must be considered, especially with co-administered drugs that share hepatic metabolic pathways or affect cytochrome P450 activity (54). Comprehensive medication review and ongoing monitoring are therefore essential to ensure both safety and therapeutic efficacy.

### Adverse effects of sertraline in the pediatric population

Sertraline is considered one of the safest and best-tolerated antidepressants within the class of SSRIs. However, its use may be associated with adverse events (AEs) that vary in frequency, intensity, and duration depending on individual patient characteristics, dosage, treatment duration, and progressively more recognized the pharmacogenetic profile of the individual (55, 56). In pediatric populations, AEs may manifest differently than in adults due to physiological factors such as immaturity of the central nervous system, developing hepatic and renal function, and variations in drug bioavailability. Therefore, close clinical monitoring during the initial treatment phase is essential (57, 58). Table 1 summarizes the most frequent and clinically significant AEs reported in children and adolescents treated with sertraline.

**Table 1:** Common and clinically significant adverse effects associated with sertraline use in pediatric populations.

Category	Adverse Effect	Clinical Comments
1. Common		
Gastrointestinal	Nausea, vomiting, diarrhea, abdominal pain, dyspepsia, loss of appetite.	Typically appear during the first weeks of treatment; tend to diminish over time or after dose reduction.
Neurological and mood-related	Headache, insomnia, somnolence, irritability, dizziness, fatigue, psychomotor agitation.	Hyperactivity or difficulty concentrating may occur; assess for possible overlap with ADHD symptoms.
Sleep disturbances	Initial insomnia, nocturnal awakenings, excessive daytime sleepiness.	Adjusting the dosing schedule may improve tolerance and sleep quality.
Appetite and weight changes	Loss or increase of appetite; mild weight fluctuations.	Generally produces less weight impact compared to other antidepressants.
2. Less frequent but clinically relevant		
Suicidal ideation or psychiatric worsening	Increased risk of suicidal thoughts or behavior, particularly at treatment onset or after dose adjustments.	Requires close monitoring; the U.S. FDA issued a safety warning in 2004.
Serotonin syndrome	Hyperthermia, rigidity, confusion, tachycardia, hypertension, hyperreflexia.	Rare but potentially serious; requires urgent medical attention; may result from overdose or interaction with other serotonergic drugs.
Sexual dysfunction	Decreased libido, anorgasmia, erectile dysfunction.	Uncommon; often underreported in adolescents.
Dermatological reactions	Rash, pruritus, urticaria.	Rare; consider discontinuation if lesions progress or affect patient comfort.

ADHD, attention-deficit/hyperactivity disorder; FDA, Food and Drug Administration.

### Common adverse effects

The most frequently reported side effects in pediatric populations primarily involve the gastrointestinal system, the central nervous system, and mood regulation. Gastrointestinal symptoms include nausea, vomiting, diarrhea, abdominal pain, dyspepsia, and loss of appetite. These typically occur during the first few weeks of therapy and tend to subside spontaneously or following dose adjustment (59). Neurological and mood-related AEs include headache, insomnia, somnolence, irritability, dizziness, fatigue, and psychomotor agitation. In some cases, hyperactivity or difficulty concentrating may occur, which can be misinterpreted as symptoms of ADHD (60). Sleep disturbances, as well as changes in appetite and body weight, have also been reported, although they are generally transient and reversible (61, 62).

### Serious adverse effects and special considerations

Although infrequent, certain sertraline-related AEs have important clinical implications and warrant close monitoring. Among these, suicidal ideation and worsening of psychiatric symptoms are the most critical in pediatric psychiatry. In 2004, the U.S. Food and Drug Administration (FDA) issued a safety alert regarding the potential increased risk of suicidal ideation or behavior in children and adolescents treated with SSRIs, including sertraline. While the absolute risk remains low, intensive clinical monitoring is recommended during the first weeks of treatment and following any dosage adjustments (63). Another rare but potentially life-threatening AE is serotonin syndrome, which may occur due to overdose or coadministration with other serotonergic agents (64). This syndrome is characterized by hyperthermia, rigidity, autonomic instability, and altered mental status, requiring immediate medical attention. Additionally, cases of sexual dysfunction have been described in adolescents treated with sertraline, as well as mild-to-moderate dermatological reactions. Although uncommon, these may necessitate discontinuation of therapy if symptoms progress or significantly affect quality of life (65, 66).

### Prevention and management of adverse events

Active monitoring during the first 2–4 weeks of treatment is essential to minimize the risk and severity of AEs. The main recommendations include:

Start with low doses and titrate gradually according to clinical response.
Conduct frequent follow-up assessments, either in person or via telemedicine, maintaining open communication with parents or caregivers.
Provide family psychoeducation, explaining potential AEs and emphasizing the importance of avoiding abrupt discontinuation.
Assess personal and family history of hypersensitivity or poor tolerance to SSRIs.
Consider pharmacogenetic testing for *CYP2C19*, *CYP2D6*, and *CYP2C9* when available, particularly in patients with unexplained adverse reactions or poor response to other antidepressants.

Early detection of AEs allows for timely interventions such as dose adjustment, modification of administration timing, or switching to another SSRI with a different pharmacokinetic profile (38, 67).

### Optimization of sertraline therapy in pediatric populations

Optimizing sertraline therapy in pediatric patients remains a clinically significant challenge, owing to the high interindividual variability in both therapeutic response and adverse event incidence. This heterogeneity arises from multiple interacting factors, including physiological maturation, clinical comorbidities, and most notably the patient's pharmacogenetic profile (48). In this context, the therapeutic objective extends beyond symptom relief to include treatment safety, minimization of adverse events, and avoidance of both underexposure and overexposure to the drug (21).

### Initial considerations for treatment optimization

Before initiating sertraline therapy, a comprehensive clinical assessment is essential. This evaluation should consider the patient's age, body weight, psychiatric diagnosis, symptom severity, comorbid medical conditions, and concomitant medications (68). In children and adolescents, it is recommended to start with low doses (e.g., 12.5–25 mg/day) and gradually titrate according to clinical response and tolerability. This stepwise strategy facilitates identification of the minimum effective dose while preventing excessive plasma concentrations and minimizing adverse effects, particularly in slow metabolizers (69).

### Role of pharmacogenetics in dose personalization

Sertraline is primarily metabolized in the liver through enzymes of the cytochrome P450 system, notably *CYP2C19*, *CYP2D6*, and *CYP2C9*, with minor contributions from *CYP3A4*. Single nucleotide variants (SNVs) in these genes result in distinct metabolic phenotypes poor, intermediate, extensive, rapid, or ultrarapid directly affecting plasma concentrations and the therapeutic profile of the drug (70, 71).

Poor metabolizers (*CYP2C19*): exhibit markedly reduced hepatic clearance, leading to higher plasma levels and an increased risk of AEs. The CPIC recommends reducing the standard dose by at least 50% or selecting an alternative antidepressant if prior intolerance has been documented (50).
Ultrarapid metabolizers: display accelerated sertraline elimination, resulting in subtherapeutic concentrations. These patients may require dose escalation or switching to another SSRI with lower dependence on *CYP2C19*-mediated metabolism (50).

The study by Poweleit et al. (2023**)** (17) in pediatric populations demonstrated that children with poor metabolizer genotypes for *CYP2C19* showed significantly higher sertraline plasma exposure, suggesting the need for individualized dosing regimens to prevent toxicity while maintaining therapeutic efficacy.

### Practical strategies for dose optimization

Implementing pharmacogenetically guided dosing strategies requires a multidisciplinary approach that integrates clinical, genetic, and pharmacokinetic information. Table 2 summarizes general dosing adjustment recommendations based on the patient's genetic profile, concomitant medications, and the presence of adverse effects or inadequate clinical response. These strategies should be applied under close psychiatric and pediatric supervision, ideally with the support of clinical pharmacogenomics services capable of interpreting genetic findings in the context of the patient's overall therapeutic plan.

**Table 2:** Recommended sertraline dose adjustments in pediatric patients according to genetic profile, comorbidities, and clinical response.

Clinical or pharmacogenetic condition	Dose recommendation/therapeutic action
Patient without available genotype	Initiate with 12.5–25 mg/day; titrate every 1–2 weeks based on tolerance and clinical response.
*CYP2C19* poor metabolizer	Reduce the initial dose by 50%; consider alternative therapy if there is a history of significant intolerance.
*CYP2C19* ultrarapid metabolizer	Consider higher doses or switching to an alternative SSRI.
Concomitant use of CYP450 inhibitors	Reduce the dose by up to 50% and monitor for signs of drug accumulation or adverse effects.
Early-onset adverse effects (e.g., nausea, somnolence, irritability)	Decrease the dose and assess hepatic function; consider extending the interval between dose increases.
Lack of clinical response after 6–8 weeks	Gradually increase the dose up to a maximum of 200 mg/day, or consider switching to another SSRI.

SSRI, selective serotonin reuptake inhibitors.

### Population pharmacokinetic modeling as a tool for therapeutic optimization

PopPK modeling represents one of the most advanced tools for optimizing pharmacotherapy in drugs with a narrow therapeutic index, such as sertraline. This approach enables the estimation of individual pharmacokinetic parameters including clearance, volume of distribution, and half-life through mathematical models that integrate plasma concentrations, demographic characteristics, and genetic variability, even when only a limited number of biological samples are available (72). In the study by Zhang et al. (2024) (21), a population-based pharmacokinetic model was developed for children with anxiety disorders in China, incorporating both pharmacokinetic and genetic parameters, including *CYP2C19* metabolic status, to design personalized dosing regimens. The model successfully optimized clinical efficacy and reduced the incidence of adverse effects, demonstrating the feasibility and clinical utility of PopPK approaches in pediatric psychiatry.

### Complementary clinical tools for therapeutic optimization

Beyond genetic testing and pharmacokinetic modeling, several complementary clinical tools can enhance the dynamic evaluation and adjustment of sertraline therapy. MDD, though not routinely applied to SSRIs, may be useful in cases of suboptimal response, suspected toxicity, or polypharmacy (73). In addition, systematic use of validated clinical rating scales such as the *Pediatric Anxiety Rating Scale (PARS)*, the *Children's Depression Inventory (CDI)*, and the *Clinical Global Impression (CGI)* allows for a quantitative assessment of symptom evolution, providing objective support for dose adjustments (74, 75). Finally, psychoeducation and adherence reinforcement are critical components of successful treatment outcomes. Active involvement of parents and caregivers throughout the therapeutic process facilitates the early detection of intolerance or adverse effects, improves treatment adherence, and strengthens trust in pharmacological management (76).

### Current limitations and future perspectives in pediatric populations

Despite the growing interest in tailoring selective serotonin reuptake inhibitor (SSRI) therapy to individual patients, the routine clinical implementation of pharmacogenetic testing and pharmacokinetic modeling remains limited in pediatric psychiatry. Economic constraints, logistical challenges, and insufficient training among healthcare professionals continue to represent major barriers to widespread adoption. In addition to these structural barriers, a critical limitation of the current evidence base is the underrepresentation of pediatric populations from diverse ethnic and geographic backgrounds, including Asian, African, and Middle Eastern regions (18–20). Differences in allele frequencies of key pharmacogenes such as *CYP2C19* and *CYP2D6* across populations may result in clinically relevant variability in sertraline pharmacokinetics, therapeutic response, and adverse event risk (10, 77). Addressing these gaps through multinational and multiethnic pediatric studies is essential to ensure equitable and globally applicable pharmacogenetically guided treatment strategies. However, with the rapid advancement of next-generation sequencing technologies and the progressive integration of personalized medicine initiatives worldwide, these tools are expected to become increasingly incorporated into standard therapeutic protocols. A key research priority involves the validation of genotype-guided dosing algorithms in well-designed, controlled pediatric clinical trials that reflect the genetic diversity of Latin American and Mexican populations. Establishing these frameworks would not only improve therapeutic outcomes but also reduce healthcare costs associated with avoidable adverse drug reactions or ineffective treatments.

### Initiating individualized therapy

Clinical practice supports initiating sertraline at 12.5–25 mg/day, with titration every 1–2 weeks based on response and tolerability. For patients weighing less than 30 kg or with a history of psychotropic hypersensitivity, even lower starting doses (6.25–12.5 mg/day) may be appropriate. Morning administration is advised to reduce the risk of insomnia. When genetic testing results are available, dosing should follow the recommendations of the Clinical Pharmacogenetics Implementation Consortium (CPIC) and the Dutch Pharmacogenetics Working Group (DPWG) (14). Table 3 summarizes genotype-based dosing adjustment strategies. For example, a child carrying the **CYP2C192/2 (poor metabolizer) genotype may exhibit elevated serum levels within the first weeks of treatment, increasing the risk of adverse effects such as somnolence, nausea, or agitation. In such cases, a 50% dose reduction at initiation, combined with close clinical monitoring, can improve tolerability without compromising efficacy. Family psychoeducation plays a pivotal role during this phase. Informing caregivers about the expected treatment course, potential transient side effects, and the importance of adherence fosters treatment continuity and reduces unnecessary discontinuations.

**Table 3:** Recommendations for sertraline dose adjustment according to *CYP2C19* phenotype. **.**

*CYP2C19* Phenotype	Pharmacogenetic Description	CPIC Recommendation (2023)	Clinical Comment
Poor Metabolizer (PM)	Markedly reduced or absent enzymatic activity; typically associated with alleles *CYP2C19* *2/*2, *2/*3, *3/*3.	Reduce the initial dose by approximately 50% or consider an alternative antidepressant if the patient has a history of intolerance or severe adverse effects.	Increased plasma exposure to sertraline and higher risk of adverse effects (e.g., somnolence, nausea, agitation). Requires close clinical monitoring.
Intermediate Metabolizer (IM)	Partially reduced enzymatic activity; commonly associated with alleles *1/*2 or *1/*3.	Initiate treatment with a standard or slightly reduced dose and titrate according to tolerance and clinical response.	Mild elevation in plasma concentrations may occur; monitor for early-onset adverse effects during the first weeks.
Extensive Metabolizer (EM)	Normal enzymatic activity; functional alleles *1/*1.	Start with the standard dose (12.5–25 mg/day) and adjust gradually based on clinical response.	Expected therapeutic response with low risk of adverse effects; standard regimen recommended.
Rapid Metabolizer (RM)	Increased enzymatic activity; associated with allele *1/*17.	Consider moderate dose increments if adequate clinical response is not achieved.	Possible subtherapeutic exposure; may require higher doses or switching to an SSRI less dependent on *CYP2C19* metabolism.
Ultrarapid Metabolizer (UM)	Significantly increased enzymatic activity; associated with allele *17/*17.	Consider an alternative antidepressant or carefully increase the dose under close clinical supervision.	Risk of subtherapeutic plasma concentrations and potential therapeutic failure.

CPIC, Clinical Pharmacogenetics Implementation Consortium; SSRI, selective serotonin reuptake inhibitors.

### Limitations of the review

Several limitations should be acknowledged when interpreting the findings of this review. First, most studies included did not involve representative samples of children under 12 years of age, restricting generalizability to this age group. Second, methodological heterogeneity across studies in design, sample size, and outcome variables precluded the performance of a quantitative meta-analysis. Additionally, the potential for publication bias cannot be excluded, as studies with negative or neutral findings are less likely to be published. Finally, there is a notable scarcity of data in Latin American and Mexican pediatric population**s**, limiting the applicability of results to local contexts and underscoring the need for region-specific research.

## Conclusion

Sertraline is among the most extensively used and clinically supported SSRIs for the treatment of anxiety disorders in pediatric populations. Despite its well established efficacy, therapeutic response and adverse event incidence exhibit high interindividual variability, representing a major challenge in clinical practice. This variability is largely attributable to hepatic metabolic differences mediated by cytochrome P450 enzymes, particularly *CYP2C19, CYP2D6*, and *CYP2C9*, whose genetic variants significantly modify sertraline pharmacokinetics. The reviewed evidence indicates that single-nucleotide variants (SNVs) in these genes directly influence plasma concentrations of sertraline and its active metabolite, thereby determining both therapeutic efficacy and adverse event risk. The application of PopPK modeling and pharmacogenetic analysis has driven advances toward a precision medicine approach in child psychiatry. This paradigm integrates genetic, physiological, and clinical data to design safer, more effective, and individualized dosing regimens, improving therapeutic outcomes while reducing adverse effects. Although the routine clinical implementation of these strategies faces logistical, economic, and training-related barriers, their progressive incorporation into pediatric mental health programs offers a promising path to enhance treatment quality and safety. Future research should prioritize controlled clinical studies in Latin American and Mexican pediatric cohorts to validate genotype-based dosing models within regional contexts. Simultaneously, it is essential to strengthen healthcare professionals' training in the interpretation of pharmacogenomic data and to facilitate access to cost-effective genetic testing integrated into public health protocols. While this review emphasizes the urgent need for pharmacogenetic and pharmacokinetic data in Latin American and Mexican pediatric populations, the concepts discussed are broadly applicable to other underserved and understudied pediatric groups worldwide. Expanding research efforts across diverse populations will be essential to achieving a truly global precision medicine framework in pediatric psychopharmacology. In conclusion, optimizing sertraline therapy in children requires an individualized and integrative approach that combines MDD with clinical, physiological, and genetic parameters. This personalized medicine framework not only maximizes therapeutic efficacy but also minimizes risks, paving the way for a safer, evidence-based pediatric psychiatry grounded in precision pharmacology.
